# Contribution of the hexosamine biosynthetic pathway in the hyperglycemia-dependent and -independent breakdown of the retinal neurovascular unit

**DOI:** 10.1016/j.molmet.2023.101736

**Published:** 2023-05-11

**Authors:** Yixin Wang, Rachana Eshwaran, Susanne C. Beck, Hans-Peter Hammes, Thomas Wieland, Yuxi Feng

**Affiliations:** 1 Experimental Pharmacology Mannheim, European Center for Angioscience, Medical Faculty Mannheim, Heidelberg University, Mannheim, Germany; 2 Division of Ocular Neurodegeneration, Institute for Ophthalmic Research, Centre for Ophthalmology, Tuebingen, Germany; 3 5th Medical Department, Medical Faculty Mannheim, Heidelberg University, Mannheim, Germany

**Keywords:** Hexosamine biosynthetic pathway, Retinal neurovascular unit, Hyperglycemia, O-GlcNAc, Endothelial cell, Pericyte

## Abstract

**Background:**

Diabetic retinopathy (DR) remains one of the most common complications of diabetes despite great efforts to uncover its underlying mechanisms. The pathogenesis of DR is characterized by the deterioration of the neurovascular unit (NVU), showing damage of vascular cells, activation of glial cells and dysfunction of neurons. Activation of the hexosamine biosynthesis pathway (HBP) and increased protein O-GlcNAcylation have been evident in the initiation of DR in patients and animal models.

**Scope of review:**

The impairment of the NVU, in particular, damage of vascular pericytes and endothelial cells arises in hyperglycemia-independent conditions as well. Surprisingly, despite the lack of hyperglycemia, the breakdown of the NVU is similar to the pathology in DR, showing activated HBP, altered O-GlcNAc and subsequent cellular and molecular dysregulation.

**Major conclusions:**

This review summarizes recent research evidence highlighting the significance of the HBP in the breakdown of the NVU in hyperglycemia-dependent and -independent manners, and thus identifies joint avenues leading to vascular damage as seen in DR and thus identifying novel potential targets in such retinal diseases.

## Highlights

• Activation of the hexosamine biosynthesis pathway (HBP) leads to breakdown of the neurovascular unit (NVU).
• Enhanced protein O-GlcNAcylation is a key feature in the pathogenesis of diabetic retinopathy.
• NDPKB deficient retina exhibits activation of the HBP, mimicking the characteristics of diabetic retina.
• Protein O-GlcNAcylation modulates molecular responses involving Ang-2, Tie2 and FoxO1 in vascular damage.
• The constituents of the HBP are therefore potential novel molecular targets to prevent the breakdown of the NVU.

## Abbreviations

AC: acellular capillary
AD: Alzheimer's disease
AGE: advanced glycation end product
Ang-2: angiopoietin 2
BBB: blood–brain barrier
BRB: blood-retina barrier
db/db: leptin-receptor-deficient mice
DME: diabetic macular edema
DON: 5-diazo-oxo-norleucine
DR: diabetic retinopathy
EC: endothelial cell
ERK: extracellular signal-regulated kinase
FoxO1: forkhead Box O1
GAPDH: glyceraldehyde 3-phosphate dehydrogenase
GCL: ganglion cell layer
GFAP: glial ﬁbrillary acidic protein
GFAT: fructose-6-phosphate aminotransferase
HBP: hexosamine biosynthesis pathway
HFD: high-fat diet
HREC: human retinal endothelial cell
IL: interleukin
ILM: inner limiting membrane
INL: inner nuclear layer
InsAkita: spontaneous mutation of insulin gene
IPL: inner plexiform layer
IRMA: intraretinal microvascular abnormalities (IRMA)
MCP: monocyte chemoattractant protein
mfERG: multifocal electroretinogram
NAD(P)H: nicotinamide adenine dinucleotide phosphate
NDPK: nucleoside diphosphate kinase
NFL: nerve fiber layer
NF-κB: nuclear factor kappa B
NLR: NOD-like receptor
NLRP3: NLR family pyrin domain-containing 3
NPDR: non-proliferative diabetic retinopathy
NVU: neurovascular unit
OCT: optical coherence tomography
OGA: O-GlcNAcase
O-GlcNAc: O-linked β-N-acetylglucosamine
OGT: O-GlcNAc transferase
OIR: oxygen-induced ischemia retinopathy
OLM: outer limiting membrane
PDR: proliferative diabetic retinopathy
RAGE: receptor of AGE
RGC: retinal ganglion cell
ROS: reactive oxygen species
Sp1: specificity protein 1
TMG: thiamet G
TNF: tumor necrosis factor
TPR: tetratricopeptide repeat
TXNIP: thioredoxin-interacting protein
UDP-GlcNAc: uridine diphosphate N-acetylglucosamine
VEGF: vascular endothelial growth factor
WT: wildtype

## 1 Introduction

The hexosamine biosynthesis pathway (HBP) is fundamental in glucose metabolism as it connects multiple essential nutrient pathways, including glucose, lipid, nucleotide, and amino acid metabolisms [1,2]. Approximately 2–5% of glucose is metabolized in the HBP and converted to uridine diphosphate N-acetylglucosamine (UDP-GlcNAc), which is further utilized to synthesize glycolipids, glycosaminoglycans, and glycoproteins [3]. The rate-limiting enzyme glutamine: fructose-6-phosphate aminotransferase (GFAT) in the HBP, is abundant in the liver, smooth muscle, kidney, retina, heart, and skeletal muscle, and regulates HBP flux through its expression and activity [4,5]. The end-product of the HBP, UDP-GlcNAc is required for the O-linked β-N-acetylglucosamine (O-GlcNAc) modification, a prevalent and often regulatory protein modification [6,7].

O-GlcNAc transferase (OGT) and O-GlcNAcase (OGA) regulate O-GlcNAc cycling by adding and removing N-acetylglucosamine (GlcNAc), respectively, from threonine and serine residues of target proteins [8]. Interestingly, these two enzymes interact with one another as well as other proteins to govern a range of cellular processes and maintain certain cellular O-GlcNAc levels [9]. OGT is expressed in all mammalian cell types [10]. It affects many cellular processes, including transcription, protein synthesis and localization, protein–protein interactions, as well as the expression and activity of transcription factors such as mammalian Sin3A (m Sin3A) and specificity protein 1 (Sp1) [[11], [12], [13]]. The proteins bind to the tetratricopeptide repeat (TPR) domain of OGT and act as bridge proteins, allowing OGT recruitment directly to its targets. Likewise, OGA is highly conserved and found to be ubiquitously present in tissues [14]. While post-translational modifications of OGA and its interactions with OGT and other proteins have been identified, the modulation of OGA activity and its substrate targeting still remain largely unknown [15].

Since the HBP has a functional significance in both physiological and pathological processes, it has been implicated in a range of diseases. HBP enzymes and O-GlcNAc cycling are universally elevated, for example, in a plethora of cancer cells. They are involved in the growth and metastasis of cancer by modulating cellular signaling pathways, transcription factors, and epigenetic changes [16]. It has been demonstrated that impaired O-GlcNAc cycling is also linked to the pathogenesis of Alzheimer's disease (AD) and Parkinson's disease [17]. Furthermore, increased glucose flux through the HBP correlates with glucose toxicity and insulin resistance in patients suffering from diabetes mellitus [18]. Numerous studies have reported that hyperglycemia results in aberrant protein O-GlcNAcylation, which is the foundation of pancreatic damage, deviant insulin synthesis, mitochondrial dysfunction, and insulin resistance [[19], [20], [21]]. Growing evidence has proposed that glucose toxicity is caused by a long-term imbalance in the interplay between O-GlcNAcylation and phosphorylation, thus altering transcription regulation [22]. Moreover, HBP enzymes and elevated protein O-GlcNAcylation are involved in the pathogenesis of diabetes-related complications [7,23].

This review will provide an overview of the HBP and O-GlcNAc cycling, discuss the impact of disordered HBP on damage of the neurovascular unit (NVU) in hyperglycemia-dependent and -independent conditions, and thus help to identify new molecular targets for the treatment of retinopathy.

## 2 HBP and the retina

**Figure 1:**
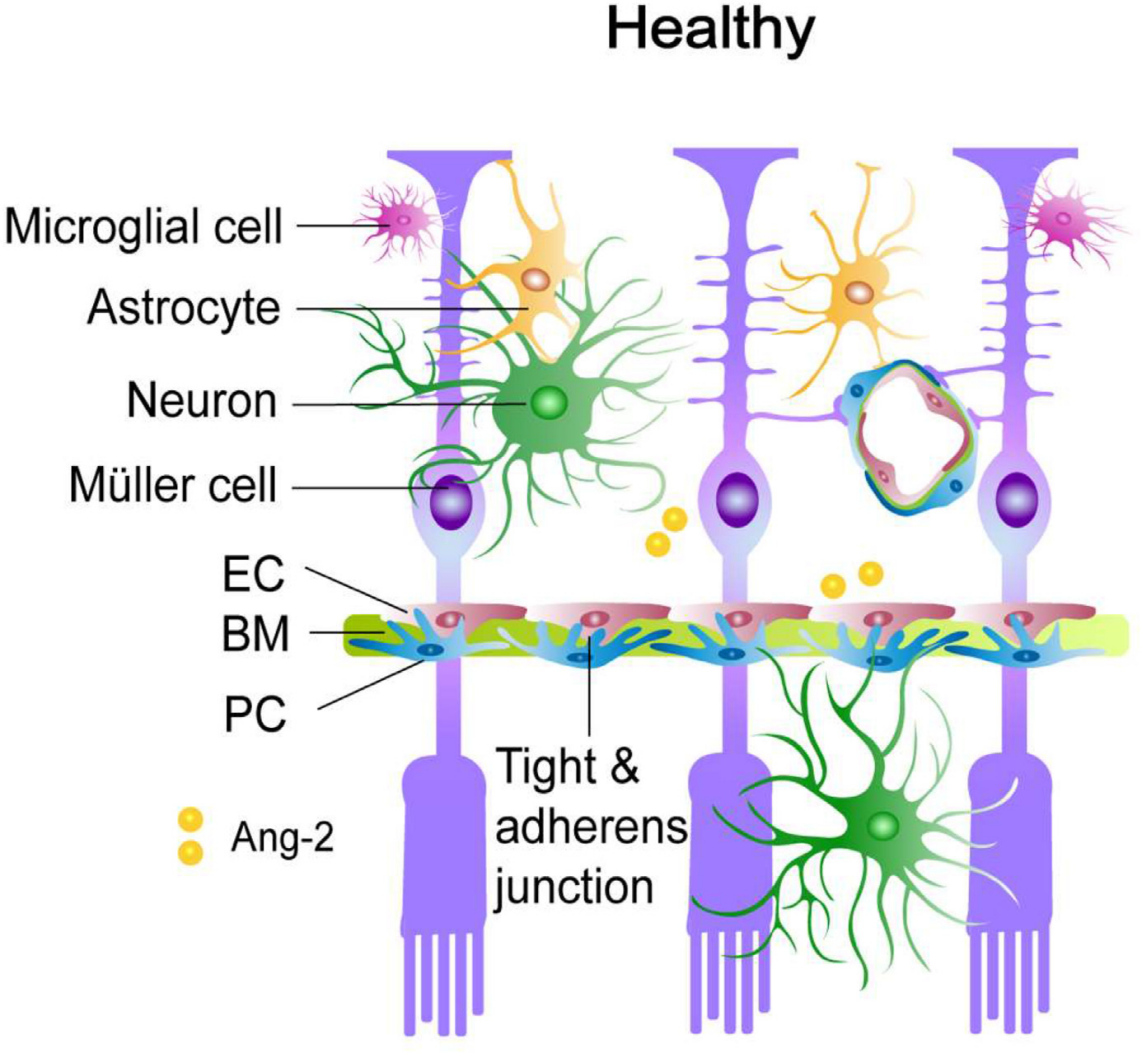
The **NVU** in health. The NVU is composed of endothelial cells (ECs), pericytes (PCs), glial cells (astrocytes, Müller cells, and microglia) and neurons. BM: basement membrane. Ang-2: angiopoietin-2.

The retina, which is a part of the central nervous system, contains a large diversity of component cells that form distinct circuits to meet the retinal metabolic requirements and maintain proper functions [24]. The retinal microenvironment is separated from the systemic circulation by the blood-retina barrier (BRB), which is structurally and functionally comparable to the blood–brain barrier (BBB) and a part of the NVU [25,26]. The NVU consists of retinal endothelial cells (ECs), pericytes, glial cells and neurons, providing an appropriate environment for the transmission of neural signals. Structurally, ECs and pericytes are embedded in the retinal vascular basement membrane, showing close ties to glial endfeet, neuronal processes, and immune cells. Appropriate synchronization of these cells modulates normal retinal function (Figure 1). An impairment of the NVU is detected in numerous retinal diseases, including DR, glaucoma, retinitis pigmentosa, cancer-related retinopathy, aging retinas, and neurodegenerative diseases [[27], [28], [29], [30], [31], [32], [33]].

Mass spectrometry has identified an abundance of proteins in the retina to be O-GlcNAc-modified, many of which are engaged in glucose metabolism [34,35]. Increasing evidence indicates that disordered protein O-GlcNAcylation is correlated to the development and progression of retinopathy mediated via mitochondrial dysfunction, increased oxidative stress, loss of neuroprotective factors and inflammation [[36], [37], [38], [39]].

## 3 HBP in hyperglycemia-dependent retinal neurovascular dysfunction - focus on diabetic retinopathy

Diabetic retinopathy (DR), a major microvascular complication of diabetes, is one of the most frequent causes of visual loss in the world, affecting approximately 100 million individuals [40,41]. DR is categorized into two stages: non-proliferative diabetic retinopathy (NPDR) and proliferative diabetic retinopathy (PDR). The features of NPDR include increased vascular permeability, microaneurysms, formation of intraretinal microvascular abnormalities (IRMA), lipid exudates, capillary non-perfusion, and neuronal impairment. PDR is distinguished from NPDR by the presence of pathologic preretinal neovascularization [42]. Diabetic macular edema (DME) can occur in both NPDR and PDR stages and typically affects working-age adults, and is characterized by exudative fluid accumulation at the macula [43,44]. DR is typically asymptomatic in the early stage, but once the disorder advances to vision-threatening DR, it can cause debilitating or permanent vision loss. Several treatment schemes have been applied clinically, such as intraocular injection of anti-vascular endothelial growth factor (VEGF) agents for DME, laser photocoagulation for PDR, and vitrectomy for vitreous hemorrhage and tractional retinal detachment. Nevertheless, these are main approaches to minimize the consequences of an already existing and severely progressed DR [29,45,46]. Therefore, preventing the onset and initial development of DR at early stages is unambiguously a good strategy for maintaining the quality of life related to vision in patients with diabetes [47].

**Figure 2:**
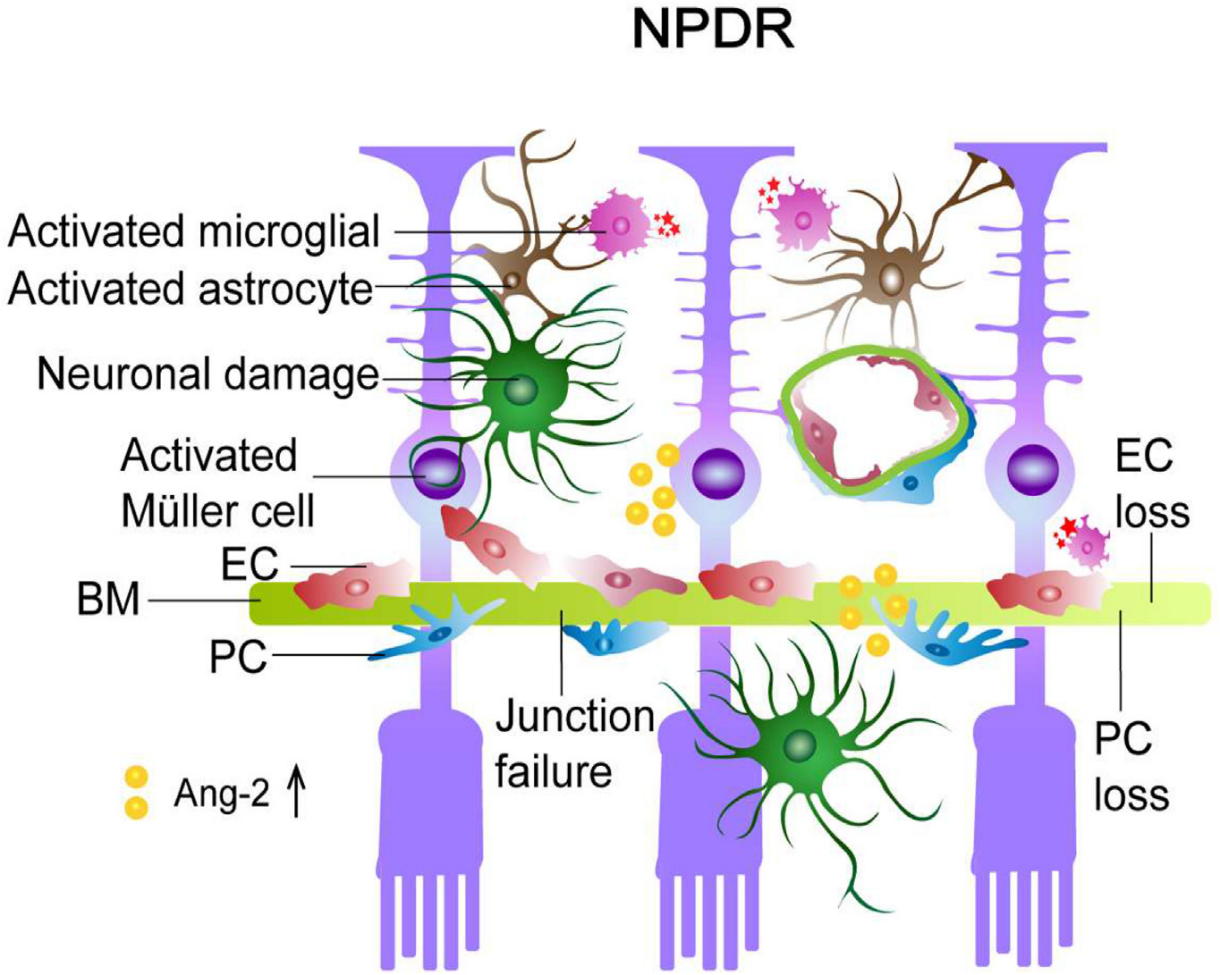
Schematic illustration of the NVU in NPDR. The diabetic retina exhibits multiple abnormalities such as loss of pericytes and endothelial cells, junction disruption and glial activation (reactive astrocytes, Müller cells and microglial cells), and neuronal dysfunction. EC: endothelial cell, PC: pericyte, BM: basement membrane, Ang-2: angiopoietin-2.

DR is a disorder of the retinal NVU, which refers to the damage of the functional coupling and interconnectivity of neurons to other NVU cellular members initiated the early stages of the disease. Hyperglycemia induces dysfunction of the retinal NVU by a breakdown of endothelial tight and adherence junctions, loss of pericytes and ECs, occurrence of acellular capillaries (ACs), glia activation and neuronal damage (Figure 2) [[48], [49], [50]]. A hyperglycemia-like vascular damage has been observed in an *in vitro* co-culture model of human BRB, in which ECs and pericytes co-cultured on opposite sides of a transwell insert and astrocytes positioned at the bottom of the culture dish were exposed to high glucose. Such hyperglycemia like conditions largely altered the permeability of the artificial BRB and interfere with adherent and tight junction proteins maintaining the BRB *in vivo* [51,52]. This will be addressed in more detail later.

Although the mechanisms driving DR development are complicated, oxidative stress likely has an important role in vascular damage [53]. Under hyperglycemia, injured retinal cells produce excessive reactive oxygen species (ROS), activating various transcription factors, and promoting critical proinflammatory signaling pathways, leading to microvascular injury [54,55]. The detrimental effect of elevated glucose in some retinal cells that ultimately results in damage to the retina is likely triggered by increased glucose flux through four major glucose metabolic pathways. The first reported pathway is the polyol pathway, followed by the pathways of advanced glycation end product and its receptor (AGE/RAGE), the HBP, and protein kinase C (PKC) activation [[56], [57], [58], [59]]. These metabolic abnormalities are interrelated, leading to neuronal and vascular dysfunctions in DR. Chronic retinal inflammation markers such as interleukin (IL)-6, IL-1β, IL-8, monocyte chemoattractant protein (MCP)-1, and tumor necrosis factor (TNF)-α, are detectable during the whole phase of DR as well as in cells treated with high glucose [60]. These important cytokines are activated by several classical pathways, including the extracellular signal-regulated kinase (ERK) and the nuclear factor κB (NF-κB) pathway, which promote the recruitment and activation of monocytes and leukocytes as well as the subsequent inflammatory responses [[61], [62], [63]]. In the 1990s, Nerlich et al. discovered that the expression of GFAT, the HBP gate-keeper enzyme, is upregulated in diabetic glomeruli, suggesting a new role of GFAT in diabetic complications [64]. Moreover, GFAT activity in diabetic subjects was significantly higher compared to control subjects, and this increased GFAT activity was correlated to postprandial hyperglycemia and insulin resistance [65]. Inhibition of GFAT was able to decrease total protein O-GlcNAc modification and restore the function of pericytes in high glucose [66].

Further research into the HBP led to the investigation of UDP-GlcNAc and the O-GlcNAc modification of proteins, and its involvement in the pathogenesis of diabetic vascular complications. Protein O-GlcNAc modification has recently been a focal point of attention of researchers to uncover the mechanisms linked to pathogenesis of DR. Due to a lack of proper human samples, the relevance of O-GlcNAc-modified proteins in the DR has been studied in animal models, including rodent diabetes models for early stages of DR, and the oxygen-induced ischemia retinopathy (OIR) model for the proliferative stages. An increase in general O-GlcNAcylation of proteins in animal models of DR, including mice with a spontaneous mutation in insulin gene (InsAkita), streptozotocin (STZ)-induced diabetic mice and rats, mice with spontaneous mutations in leptin receptors (db/db), and mice fed with high-fat diet (HFD), was observed [[66], [67], [68], [69]]. It occurs as early as six weeks of age in diabetic InsAkita mice, at two months after hyperglycemia in STZ-induced diabetic rats, and during the active neovascularization phase in the OIR mice [66,70]. Enhanced protein O-GlcNAcylation occurs predominantly in the ganglion cell layer (GCL), the inner nuclear layer (INL), and the inner plexiform layer (IPL) [69] and O-GlcNAcylated proteins co-localize with markers of retinal blood vessels in rodent DR models. This enhanced O-GlcNAc modification of target proteins results in severe alterations in the transcriptional and translational regulation of gene expression [36]. In addition, the HBP is involved in hyperglycemia-induced NF-κB activation as the O-GlcNAcylation of NF-κB p65 and its transcriptional activity was found to be increased in STZ-induced diabetic mice [71]. Moreover, in the InsAkita mice, the expression of OGT and OGA increased predominantly in the INL in diabetic mice. The expression of OGT was dramatically increased in diabetic retinas, while OGA was substantially less upregulated than OGT in the diabetic InsAkita mice [72]. In STZ-induced diabetic mice, upregulated OGT and decreased OGA in retinas were shown by Gurel et al. [68]. Therefore, not only the HBP but also downstream O-GlcNAc cycling is activated, indicating the importance of the HBP and protein O-GlcNAcylation in DR.

## 4 HBP and retinal ECs

Retinal ECs are the main component of the BRB and constitute the inner lining of the retinal vasculature. They are also the first retinal cell type to encounter hyperglycemia, since the energy production in ECs relies predominantly on glucose. The morphological and physiological properties of ECs are congruent with nutritional needs [73]. Retinal ECs are in charge of maintaining retinal homeostasis by transporting oxygen, fluids, nutrients, metabolites, and ions. Additionally, many other factors contributing to pathological processes, such as leaky blood vessel and neovascularizations in the diabetic retina, are highly correlated with EC damage. Thus, retinal ECs are one of the most important players in the onset and progression of DR [74].

It has been hypothesized that the damage of the vascular endothelium is the fundamental defect in DR, and that the consequence may be termed a “endotheliopathy.” [75]. Retinal ACs form as a result of the loss of pericytes and ECs, which is a basic premise of progressive ischemia and a leading cause of BRB failure in DR [76,77]. In long-term diabetic animal models, it is a common observation that retinal vessels degenerate. These ACs are not perfused, appearing as bare basement membrane tubes, which are also called ghost capillaries [78,79]. The underlying mechanism of the loss of retinal ECs is predominantly apoptosis of ECs in response to stressors such as hyperglycemia and oxidative stress. Continuous overproduction of ROS induces apoptosis in retinal ECs by activating caspase-3, NF-κB, and other transcription factors that speed up the death of ECs [80]. Gurel et al. demonstrated a retina-specific endothelial upregulation of protein O-GlcNAcylation under hyperglycemia, providing evidence that hyperglycemia-dependent HBP activation promotes the O-GlcNAc modification of endothelial proteins, thereby leading to endothelial dysfunction in DR [66,81].

The two key junctional structures maintaining the integrity of the ECs are adherens junctions and tight junctions. VE-Cadherin is an important adherens junction protein on ECs, and it promotes homotypic endothelial cell–cell adhesion, which supports the maintenance of vascular integrity and key functional properties of ECs [82]. Rangasamy et al. found that VE-cadherin is already phosphorylated to some extent in normal conditions, but high glucose treatment markedly increased its phosphorylations in human retinal endothelial cells (HRECs) which is also observed in diabetic retinas. These data point to the significance of VE-cadherin and its phosphorylation status for the maintenance of the integrity of the retinal EC barrier under physiological and pathological conditions [83].

Using a modified model to mimic the inflammatory conditions under hyperglycemia, Lenin et el. showed a disruption of the continuous and zigzag expression pattern of VE-cadherin at the plasma membrane, concomitant with a significant increase in tyrosine phosphorylation of VE-Cadherin, in response to high glucose and TNFα in ECs [84]. Furthermore, VE-Cadherin can be O-GlcNAcylated, and its O-GlcNAcylation increased in high glucose conditions, as well as by treatment with an OGA inhibitor. The elevation of O-GlcNAc by an OGA inhibitor also mimicked high glucose conditions with regard to tyrosine phosphorylation of VE-cadherin. In accordance, the reduction of O-GlcNAc by OGT inhibition ameliorated VE-cadherin phosphorylation. The data from these studies thus indicate that the increased protein O-GlcNAcylation under hyperglycemia can contribute to vascular hyperpermeability via the modulation of VE-cadherin phosphorylation [85].

Over the last decades, VEGF has been established as a potent inducer of vascular permeability *in vivo*. Marked VEGF immunoreactivity was observed in the diabetic retinal capillaries in the outer part of the INL [86]. Peters et al. demonstrated that VEGF modulates the integrity of ECs and hence increases vascular permeability [87]. VEGF promotes a significant time-dependent increase in permeability, which correlates to a strong induction of tyrosine phosphorylation of adherens junction components, especially VE-cadherin, in ECs [88]. Indeed, VEGF-induced permeability is mediated via Y685 phosphorylation and subsequent internalization of VE-cadherin [89]. Of note, previous *in vitro* studies showed that high glucose treatment greatly elevates the levels of VEGF mRNA and protein in ECs [[90], [91], [92]]. An increase in O-GlcNAcylation by OGA inhibitors also elevates VEGF expression, while, inversely, a decline in O-GlcNAcylation via OGT inhibition abolishes high glucose-prompted VEGF expression. Furthermore, upregulation of VEGF under hyperglycemia in preclinical DR appeared to be mediated via enhanced binding of the O-GlcNAcylated transcription factor Sp1 to the VEGF promoter, suggesting an essential role of O-GlcNAcylation in the regulation of growth factors in DR [93].

Numerous studies indicate that another growth factor, i.e. angiopoietin 2 (Ang-2), regulating the angiopoietin–Tie2 signaling pathway, is involved in endothelial–endothelial and endothelial–pericyte communications. Tie2 is a receptor tyrosine kinase, and both ligands, angiopoietin 1 (Ang-1) and Ang-2, bind to the same site in its extracellular domain. Tie2 is predominantly expressed by ECs and displays a dual role in vascular stability and integrity depending on its ligand binding status [94,95]. Ang-1 is a full agonist for Tie2 phosphorylation that contributes to vascular stabilization and decreases vascular permeability during its remodeling [96]. Ang-2 is an antagonistic ligand of Tie2, and it competitively inhibits the binding of Ang-1 to Tie2, leading to vascular destabilization and hyperpermeability. Thus, the overexpression of Ang-2 mimics Ang-1 or Tie2 deficiency [97]. Both Ang-1- and Tie2-deficient mice die due to severe defects in vascular remodeling. ECs are one of the main sources of Ang-2 in the retina and can release Ang-2 under stress conditions [83,[98], [99], [100]]. Levels of Ang-1 and Ang-2 in vitreous humor from patients with NPDR and diabetic macular edema are significantly elevated compared with controls [101]. In the diabetic rat retina, Ang-1 and Ang-2 are persistently upregulated by hyperglycemia. Notably, Ang-2 is upregulated to a larger extent than Ang-1, suggesting that Ang-2 is a key regulator in the initiation of vascular dysfunction in DR [102].

A previous study showed that the overexpression of Ang-2 in the retinas of non-diabetic mOpsinhAng2 mice induces the formation of ACs comparable to diabetic wildtype (WT) mice [103]. In agreement, Ang-2 deficiency decelerates hyperglycemia-induced formation of ACs in the retina [104]. Additionally, the administration of recombinant Ang-2 into normal rat eyes increases retinal vascular hyperpermeability nearly 3-fold compared to control animals. Concomitantly, the increased phosphorylation of VE-cadherin is associated with the vascular hyperpermeability induced by Ang-2 [83]. Peters et al. further reported that Ang-2 and VEGF control vascular permeability synergistically [87].

Ang-2 in ECs under high glucose is regulated by multiple path*ways*. Firstly, Ang2 in ECs is controlled by its receptor Tie2. Studies have detected an increased expression of Ang-2 in ECs in which Tie2 is abundant when treated with high glucose. Scharpfenecker and colleagues reported a mechanism of Ang-2 upregulation through an autocrine loop, in which secreted Ang-2 by ECs disrupts the integrity of the EC monolayer. The Ang-2-induced rapid endothelial destabilization was rescued by Ang-1, VEGF, or soluble Tie2 as Ang-2 scavenger [105]. Secondly, expression of Ang-2 can be controlled by transcription factors. The forkhead box protein transcription factor FoxO1 plays a key role in high glucose-induced gene regulation in ECs in a transcriptional and posttranslational manner. Diabetes elevates FoxO1 mRNA expression, its nuclear translocation, and DNA binding activity in the retina. The inhibition of FoxO1 reduces hyperglycemia-evoked apoptosis of ECs [106]. Increased nuclear FoxO1 is linked to a significant increment in the expression of FoxO1-regulated proteins, including Ang-2, and the upregulation of Ang-2 is considered to be mediated via FoxO1-Akt signaling [107]. The siRNA-mediated depletion of FoxO1 resulted in a decrease in Ang-2 in Weibel-Palade bodies, suggesting that FoxO1 is indeed involved in the regulation Ang-2 in ECs [108]. Several domains, especially the phosphorylation sites in FoxO1 are essential for the FoxO1 shuttling between nucleus and cytosol [109]. Studies have also shown that FoxO1 can be O-GlcNAcylated in normal and hyperglycemic conditions [110]. High glucose-enhanced O-GlcNAcylation of FoxO1 on the same residues targeted by phosphorylation, reduces FoxO1 phosphorylation, causing translocation of FoxO1 into the nucleus, increasing the ratio of FoxO1 between the nuclear and cytosolic fractions [111]. Moreover, Ang-2 is noticeably O-GlcNAcylated in normal ECs, and increased protein O-GlcNAcylation evoked by the treatment with OGA-specific inhibitor Thiamet G (TMG) or OGA knockdown significantly elevated Ang-2 content in ECs, indicating that Ang-2 levels in ECs are susceptible to changes in cellular O-GlcNAcylation [112]. Yao et al. discovered a novel mechanism for Ang-2 upregulation by activation of the HBP under high glucose in ECs. The transcription factor Sp3 is greater O-GlcNAcylated in high glucose condition. Enhanced O-GlcNAc modification of Sp3 results in the formation of a complex with Sp3, corepressor m Sin3A and OGT, causing decreased binding to a glucose-responsive GC-box in the Ang-2 promoter, leading to increased Ang-2 expression [113].

Interestingly, studies also show that increased protein O-GlcNAcylation in ECs exposed to various stresses including oxidative stress might play a critical role in cellular protection processes. Increased cellular O-GlcNAcylation is able to protect protein structure and function against damage among dysregulation of protein activity as a survival response [114]. Additionally, increased O-GlcNAcylation is capable of protecting retinal ECs against ROS in high glucose conditions. The enhancement in O-GlcNAcylation acts in a cytoprotective manner on ECs by lowering ROS production, boosting the expression of antioxidant genes, limiting mitochondrial membrane potential dissipation, and preventing EC apoptosis [115].

## 5 HBP and retinal pericytes

Pericytes play a key role in the stability, homeostasis, and contractility of capillaries [116]. They are implanted in the basement membrane surrounding ECs, extend along and around pre-capillary arterioles, capillaries, and post-capillary venules, and cover numerous ECs and the associated junctions between ECs. The topographical density of pericytes in the retina is similar to ECs, higher than in other tissues [117]. They are essential cellular constituents of the NVU in the retina and serve to maintain the BRB in physiological and pathological conditions such as hyperglycemia [118,119]. Chronic hyperglycemia has been proven repeatedly to have a fundamental impact on pericytes and their interaction with adjacent coupling cells. The intercellular communication between pericytes and ECs is dramatically reduced in the STZ-induced diabetic rat retina [120].

Notably, the extent of protein O-GlcNAcylation in distinct retinal vascular cells varies under different circumstances. Pericytes were shown to have the lowest basal levels of protein O-GlcNAcylation compared to ECs and astrocytes. However, protein O-GlcNAcylation in pericytes responds to high glucose to a greater extent than in ECs. High glucose or OGA inhibitors increases total O-GlcNAc modification in retinal pericytes, whereas OGT inhibitors or GFAT inhibitors under high glucose suppresses overall O-GlcNAc modification in retinal pericytes. Hyperglycemia-promoted protein O-GlcNAcylation in the pericytes is seen to be a result of decreased OGA expression while the expression of OGT is unchanged [66]. At the early stages of diabetes, pericytes are the most sensitive cell type, and disappear first under hyperglycemia [121].

Pericyte loss is a characteristic feature of DR, and changes in pericyte function exist already at the early stages of DR. Upon pericyte loss, ECs begin to disappear from the microvasculature and the remaining empty basement tubes cannot be perfused. They are finally filled with glial end feet [122]. The main causes of pericyte loss in DR are pericyte apoptosis and pericyte migration. Pericyte apoptosis was observed in animal models of DR as well as *in vitro* under high glucose conditions [106,123]. Multiple signaling pathways are involved in pericyte apoptosis in the retina. Elevated AGE in high glucose induces activation of caspase-3 [124]. Suarez et al. reported that pericyte apoptosis caused by high glucose is mediated by the glyceraldehyde 3-phosphate dehydrogenase (GAPDH)/Siah1 pathway [125]. Moreover, Cacicedo et al. demonstrated that pericyte apoptosis is linked to a high glucose-induced disorder in the saturated fatty acid palmitate via oxidative stress and activation of nicotinamide adenine dinucleotide (NAD(P)H) oxidase and NF-κB [126]. In *in vitro* studies, high glucose induces the expression of the thioredoxin-interacting protein (TXNIP), a pro-oxidative stress and pro-apoptosis protein, causing enhanced oxidative stress, mitochondrial dysfunction, ATP depletion, DNA damage, and apoptosis in pericytes [127]. High glucose-induced pericyte apoptosis can be protected by the treatment with an anti-oxidant, as well as TXNIP knockdown by siRNA via suppressing ROS production, caspase-3 activation, and DNA damage [127]. These results suggest that oxidative stress plays a pivotal role in pericyte apoptosis [128]. Pericyte apoptosis is linked to protein O-GlcNAcylation as well. Treatment with the OGA inhibitors TMG and PUGNAc, the first generation of OGA inhibitors, both known to enhance protein O-GlcNAcylation, suppressed retinal PC survival under normal glucose conditions. Reversely, 5-diazo-oxo-norleucine (DON) and alloxan, two compounds reducing protein O-GlcNAcylation, ameliorated the deleterious effect of high glucose on the survival of retinal pericytes [81]. All these findings indicate that hyperglycemia-induced protein O-GlcNAcylation is a driver of pericyte damage in the retina. Furthermore, many proteins involved in the process of programmed cell death are O-GlcNAcylated in pericytes under hyperglycemia [81]. Reports have identified p53 as an O-GlcNAcylation target, and a potential key protein in pericyte apoptosis. p53 is O-GlcNAcylated at Ser149, interfering with the phosphorylation at Thr155 in high glucose conditions, further regulating p53 activity and stability [129]. Levels of O-GlcNAc-modified p53 are elevated in pericytes cultured in high glucose conditions, and an elevated O-GlcNAcylation of p53 was found to contribute to selective initial loss of pericytes in diabetes [81].

As mentioned above, FoxO1, a key regulator of cell proliferation, survival, and apoptosis, is also associated with pericyte apoptosis in the diabetic retina. Alikhani et al. revealed that apoptosis induced by TNF-α and AGE in retinal pericytes is mediated by the activation of FoxO1, contributing to the pathogenesis of DR [130]. FoxO1 knockdown lowers not only the AC number but also pericyte loss in STZ-induced diabetic animals [90]. The reduction of hyperglycemia-induced pericyte apoptosis by the suppression of FoxO1 occurs through the inhibition of caspase-3 [106,130]. Although it has been found that FoxO1 is modified by O-GlcNAcylation, studies on GlcNAcylated FoxO1 in retinal pericytes under hyperglycemia are missing [108,131].

Recent studies have also reported a novel mechanism of pericyte loss - pericyte migration in the diabetic retina [104,132,133]. Ang-2 upregulation was found to be associated to a great extent with pericyte migration and pericyte loss [104]. The number of migrating pericytes increases dramatically in diabetic retinas after 6 months of hyperglycemia compared with nondiabetic controls. While pericytes on capillary branches are fully unaffected by chronic hyperglycemia, pericytes on straight capillaries get perceptibly lost. Ang-2 is upregulated in diabetic retinas prior to pericyte loss [102]. Experiments with genetic manipulation - gain and loss of function of Ang-2 – provide evidence for the essential role of Ang-2 in pericyte loss at the early stage of DR. *ANG-2* gene haplo-deficiency eliminates hyperglycemia-induced pericyte loss in the Ang-2LacZ retinas. Overexpression of Ang-2 in mOpsinhAng2 mice mimics the diabetic WT retinas, showing promoted pericyte migration and loss [103,104]. Furthermore, in diabetic mOpsinhAng2 retinas, the migration of pericytes is aggravated compared with their non-diabetic controls [104]. Notably, Gurel et al. found that the migration of retinal pericytes *in vitro* is decreased by high glucose, and inhibition of HBP activity reverses migrating properties of retinal pericytes [81]. Apparently, the migration of pericytes thus differs *in vivo* from that *in vitro*. Also, the underlying mechanisms of pericyte migration correlated to protein O-GlcNAcylation need further investigation.

## 6 HBP and retinal glial cells

The rodent retina comprises two types of glial cells – macroglia, including astrocytes and Müller cells, and microglia, which are components of the NVU. Glial cells, together with pericytes, restrict EC growth and maintain vascular stability of the BRB. Various glial homeostatic activities, such as carbon dioxide removal, extracellular pH adjustment, and clearance of excess potassium from the extracellular fluid, are carried out by retinal astrocytes as well as Müller cells. Glial cells play critical roles in retinal blood flow, vascular permeability, and cell survival [134,135].

Astrocytes are located mostly in the nerve fiber layer (NFL) and the GCL [136]. Astrocytes can be activated and overexpress glial ﬁbrillary acidic protein (GFAP) after retinal damage. *In vitro* analysis revealed that Ang-2 triggers astrocyte apoptosis, particularly at high glucose concentrations. In STZ-induced diabetes mice, the Ang-2-induced loss of retinal astrocytes by apoptosis can be prevented by intravitreal injection of an Ang-2-neutralizing antibody [137]. There are few studies addressing protein O-GlcNAcylation in glial cells. Astrocytes exhibit the highest level of O-GlcNAcylation among retinal ECs, pericytes, and astrocytes. Nevertheless, protein O-GlcNAcylation in astrocytes remained unchanged under high glucose conditions. Thus, astrocytes are relative resistant to high glucose treatment [81]. Interestingly, not only the levels of protein O-GlcNAcylation, but also those of OGT and OGA are higher in astrocytes compared to ECs and pericytes in the retina [66]. Functional consequences of these features are not yet known.

Müller cells are specialized radial glia that cover the entire thickness of the neural retina between the inner (ILM) and outer limiting membrane (OLM) [138]. Müller cells offer homeostatic, metabolic, and functional support to neurons, and are involved in the structural stability of the retina and in immunological and inflammatory responses. Pathogenic stimuli causing activation of Müller cells are presumed to play an essential role in neuroprotection [139]. Both hyperglycemia and hypoxia can harm Müller cells and promote a breakdown of the NVU in diabetes [139]. Müller cells are activated and produce excessive GFAP in STZ-induced diabetic animals at the early stage of DR [98,140]. Besides ECs, macroglia is the largest source of Ang-2 in hyperglycemic conditions in the retina [98]. The HBP-activation controlled upregulation of Ang-2 via m Sin3A described in the previous paragraph “HBP and retinal ECs” is apparently a common mechanism to ECs and Müller cells. In both cell types in the retina, high glucose promotes Ang-2 expression via O-GlcNAc modification of transcription factors [113]. Some studies have reported the link between protein O-GlcNAcylation and glial activation. Hyperglycemia induces enhanced O-GlcNAcylation of some translation initiation factors including 4 E-BP1 [141]. CD40, a member of the TNFR superfamily, was found to be upregulated by hyperglycemia in retinal Müller cells. Hyperglycemia-induced CD40 expression in Müller glia is mediated via enhanced O-GlcNAcylation of 4 E-BP1/2, leading to neurovascular degeneration in DR [142]. The gap junction protein Cx43 can be modified by O-GlcNAcylation as well [143]. Hyperglycemia upregulates Cx43 expression in glial cells, causing activation of glial cells. Inhibition of protein O-GlcNAcylation dramatically reduces Cx43 expression and glial activation induced by hyperglycemia [70], indicating that O-GlcNAcylation of certain key proteins may contribute to the development of DR despite the fact that they are fundamentally insusceptible to cellular O-GlcNAcylation levels.

## 7 HBP and retinal neurons

Concurrently, an increasing amount of data demonstrates that the damage of neurons occurs in diabetic and prediabetic retinas, preceding vascular closure and it accelerates vision loss [144,145]. Using optical coherence tomography (OCT), a loss of retinal ganglion cells (RGCs) has been observed in diabetic individuals without visible microvascular lesions, and this neuronal damage progresses with the onset of moderate or severe DR [146]. The RGC damage evoked by hyperglycemia contributes to a reduction in the thickness of the retinal NFL in the macula of diabetic eyes even when there are no clinical signs of retinopathy [147]. By multifocal electroretinogram (mfERG) a considerably increased loss of neuronal function was detected in diabetic patients with retinopathy than in those without. The mfERG anticipates the sites of matching retinal malfunction where patients develop retinopathy in the next 1–3 years [148,149]. However, there are very few studies addressing the link between the HBP and neuronal damage in DR. Seong-Jae et al. explored protein O-GlcNAcylation expression in neurons and found that O-GlcNAc in the GCL and INL is higher in diabetic mice retinas than in controls and nuclear translocation of the p65 subunit of NF-κB is obviously increased simultaneously. Immunoprecipitation experiments showed that NF-κB p65 subunit is O-GlcNAcylated, and it contributes to RGC death in DR [68]. Furthermore, Nakamura et al. showed that high glucose inhibit the anti-apoptotic effect of insulin in retinal neurons via HBP activation *in vitro* [39]. Interestingly, supplementation of glucosamine, an intermediate metabolite of the HBP, was shown to have a similar detrimental effect on neurons [39]. Nevertheless, a neuroprotective effect of exogenous glucosamine supplementation was reported in diabetic retinas *in vivo* [150]. Taken together, the relationship between the HBP and neuronal damage in the diabetic retina remains largely unexplored and needs further clarification

## 8 HBP in hyperglycemia-independent retinal neurovascular dysfunction – focus on the NDPKB deficient retina

**Figure 3:**
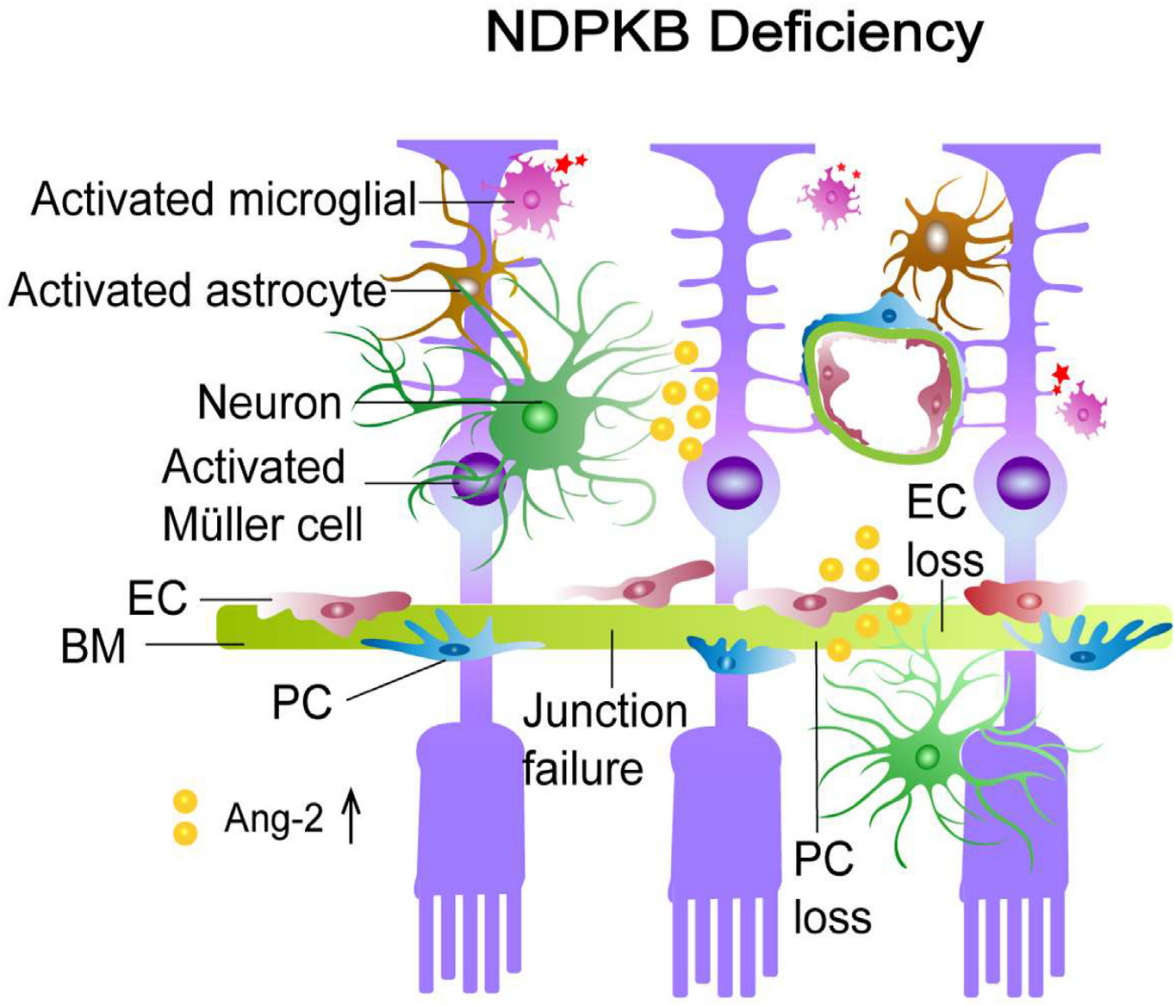
Schematic illustration of the NVU in NDPKB deficient retina. In NDPKB deficient mice, similar retinal abnormalities to DR appear, including loss of pericytes and endothelial cells and activation of glial cells. EC: endothelial cell, PC: pericyte, BM: basement membrane, Ang-2: angiopoietin-2.

Nucleoside diphosphate kinases (NDPKs) are housekeeping enzymes primarily involved in nucleotide homeostasis, facilitating the transfer of the γ-phosphate between nucleotides by employing a ping-pong mechanism and the formation of a phosphohistidine intermediate [151]. In the last decade, one member of the family of NDPKs, namely NDPKB, has been shown to be crucial for the maintenance of normal vascular function [152,153]. Deficiency of NDPKB in the retina and ECs leads to abnormal angiogenesis mediated by VEGF receptor 2 and the endothelial adherens junction protein VE-cadherin [152]. Non-diabetic NDPKB^−/−^ mouse retinas at 8 months of age display similar characteristics of retinal vascular morphology to diabetic WT retinas, such as loss of pericytes and formation of ACs. Furthermore, diabetes induced by STZ treatment exacerbates the vascular damage in NDPKB^−/−^ mice by decreasing the coverage of pericytes and enhancing the number of AC considerably [153]. Based on the primary feature of pericytes and ECs in the stabilization and survival of retinal vasculature, these results suggest the possible role of NDPKB as a protective factor in the retina [153]. Moreover, Qiu et al. demonstrated the presence of reactive astrocytes and Müller cells in the NDPKB^−/−^ retinas, a major source of excessive Ang-2, forcing pericyte loss in the retina [98]. Concomitantly, activation of microglial cells much like in DR was also detected in 5-month NDPKB^−/−^ retinas using a CD74 antibody, a novel marker of activated microglial cells (yet unpublished data). Nevertheless, *in vivo* analysis of retinal morphology and thickness using high resolution OCT imaging did not show any alterations in NDPKB^−/−^ mice compared to WT animals. Furthermore, neuronal dysfunction was not found in the full-field ERG under both scotopic and photopic conditions in the 5-month NDPKB^−/−^ retinas (yet unpublished data). The neuronal function in NDPKB^−/−^ retinas was not assessed in elder mice, in which its dysfunction might appear. On the other side, it cannot be excluded that NDPKB deficiency may trigger neuroprotective pathways as well. Nevertheless, NDPKB^−/−^ retinas exhibit a loss of pericytes, the formation of ACs, and the activation of glial cells, largely mimicking DR (Figure 3).

Previous publications have demonstrated mechanisms underlying the diabetes-like retinas in NDPKB^−/−^ mice. Surprisingly, non-diabetic NDPKB^−/−^ retinas show elevated Ang-2 levels comparable to the diabetic WT retina, significantly higher than those in non-diabetic WT controls. Furthermore, enhanced Ang-2 expression has been identified in NDPKB-depleted HUVECs *in vitro*, which is comparable to high glucose conditions, similar to the data from NDPKB^−/−^ retinas [153]. Just 50% deficiency in Ang-2 gene using heterogeneous Ang2LacZ mice crossbred into the homozygous NDPKB^−/−^ mice results in abolishment of pericyte loss and formation of ACs induced by deficiency of NDPKB [108].

Several signaling pathways appear to contribute to the regulation of Ang-2 and subsequent vascular damage in NDPKB deficient conditions. An Ang-2-Tie2 feedback loop has been reported to cause NVU injury in NDPKB deficient retinas. Concomitant with the upregulated Ang-2 in NDPKB^−/−^ retinas, the expression of Tie2 increases in NDPKB^−/−^ retinas compared to WT retinas, predominantly in the retinal deep capillary layer where the onset of vascular damage and degeneration commences. The upregulation of Ang-2 is also linked with the increase in Tie2 levels as a common phenomenon in NDPKB-depleted ECs. Concurrently, an enrichment of Tie2 at the plasma membrane was observed in NDPKB-depleted ECs. The phosphorylation of Tie2, reflecting the proportion of activated receptors, decreases upon NDPKB depletion despite elevated total membranous Tie2 expression. Interruption of the binding of Ang-2 to membranous Tie2 using soluble Tie2Fc triggers an abrogation of Ang-2 upregulation in NDPKB-depleted ECs. Ablation of Tie2 mediated by Tie2 siRNA in NDPKB-depleted ECs suppresses effectively the upregulation of Ang-2. Simultaneously, adenoviral overexpression of extracellular Ang-2 promotes Tie2 upregulation in ECs. The data suggest that the upregulation of Ang-2 in NDPKB knockdown ECs is mediated by membranous Tie2. Overall, a positive feedback loop via the Ang-2-Tie2 interaction is involved in NDPKB deficiency-induced injury of the NVU [154]. Whether Tie2 itself can be modulated by the HBP, e. g. by O-GlcNAcylation in the absence of NDPKB, requires further investigation.

Furthermore, Qiu et al. examined the relevance of the protein O-GlcNAcylation in NDPKB deficient conditions. In non-diabetic NDPKB^−/−^ retinas and in NDPKB-depleted ECs, protein O-GlcNAcylation is elevated to the extent seen in diabetic WT retinas, and in ECs stimulated with high glucose [153]. Since Ang-2 upregulation was shown to be correlated with the O-GlcNAc cycle, Chatterjee, A. et al. examined its relevance in the activation of the HBP upon NDPKB deficiency. The end product of the HBP, UDP-GlcNAc is elevated in NDPKB-depleted ECs but not in NDPKB^−/−^ retinas, implying that NDPKB deficiency may trigger HBP activation in a cell type-specific manner. The key enzyme in the HBP, GFAT1, was found to be activated in NDPKB-depleted ECs. Interestingly, despite activation of the HBP, the glycolysis and mitochondrial respiration in the NDPKB-depleted ECs are unchanged, signifying that the alteration is HBP-specific, and it does not involve the entire cellular glucose metabolism in NDPKB deficient conditions. Further, the two key enzymes in the O-GlcNAc cycle were assessed. OGA enzyme activity is significantly lower in NDPKB deficient ECs, although the protein levels of OGT and OGA are unchanged compared with controls. The results thus indicate that cellular protein O-GlcNAcylation control the Ang-2 content in NDPKB deficient conditions [112].

Moreover, another study by Shan et al. showed that increased protein O-GlcNAcylation in NDPKB-depleted ECs facilitated the O-GlcNAcylation of FoxO1. Protein O-GlcNAcylation is enhanced significantly in the cytoplasm and the nucleus. FoxO1 protein is simultaneously upregulated, similar to Ang-2 upregulation, localized in the cytoplasm as well as the nucleus in NDPKB-depleted ECs [108]. O-GlcNAcylation inhibitors reduce the expression of FoxO1 and Ang-2 in NDPKB-depleted ECs. Hence, the augmentation of FoxO1 upon NDPKB depletion is dependent on its O-GlcNAcylation, inducing the expression of Ang-2. The finding of the study by Shan et al. highlights the significance of HBP-mediated activation of the transcription factors in the regulation of Ang-2 [108].

In addition, Ang-2 itself was found to be O-GlcNAcylated in normal ECs and increasingly modified by O-GlcNAc along with elevated Ang-2 levels in NDPKB-depleted conditions. Yet whether and how the increased O-GlcNAcylation of Ang-2 upon HBP activation contributes to vascular damage, in particular, pericyte loss and formation of ACs in NDPKB deficient conditions still remain unclear.

## 9 Conclusion

In this review, we summarized the current findings on the impact of the HBP and O-GlcNAcylation on neurovascular dysfunction in hyperglycemia-dependent and -independent manners.

**Figure 4:**
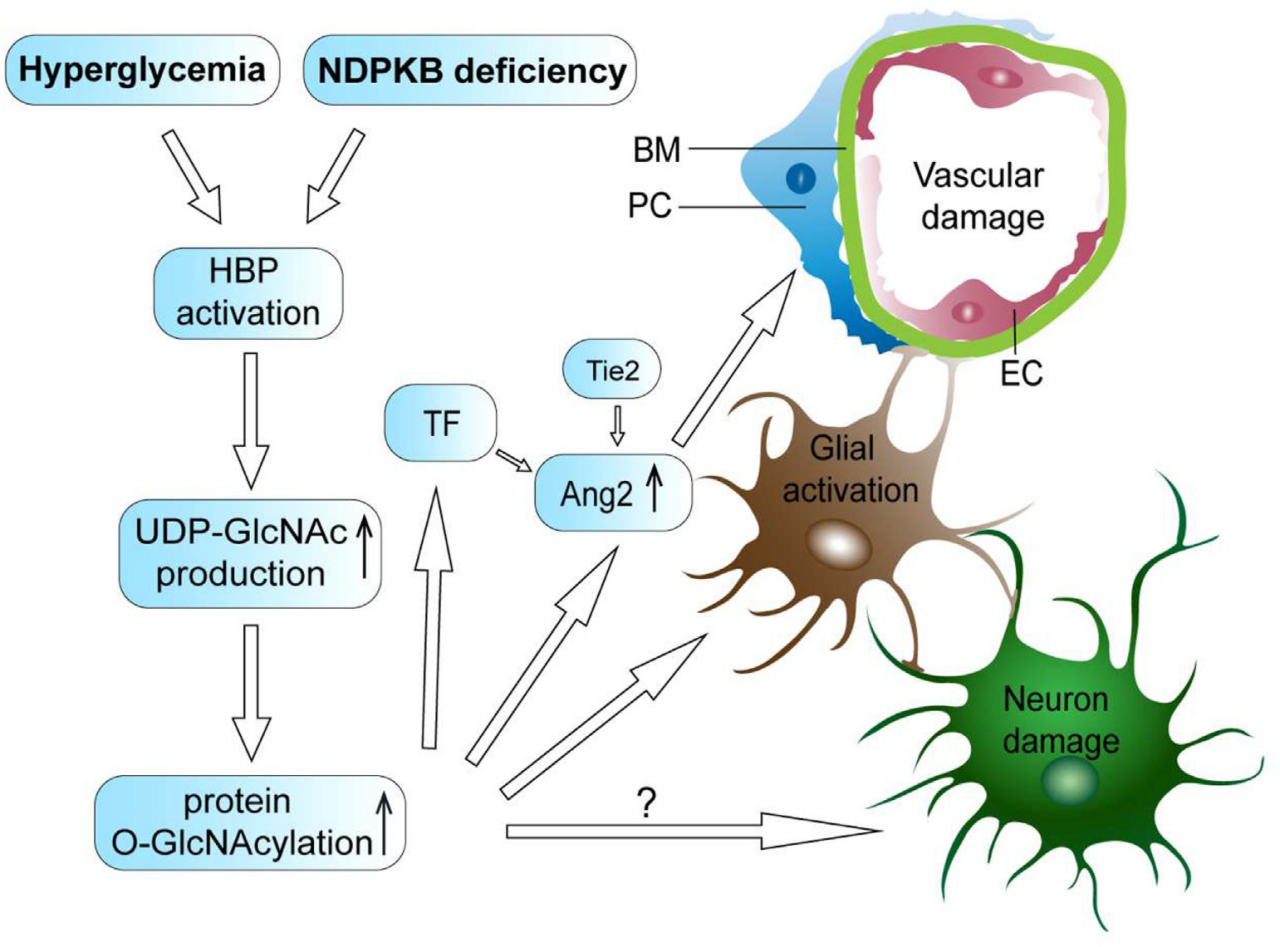
Illustration of the NVU damage in diabetic and NDPKB deficient retinas. Hyperglycemia or NDPKB deficiency activates the HBP. Increased HBP flux results in an increment of UDP-GlcNAc production and subsequent O-GlcNAcylated proteins, leading to the upregulation of Ang-2 which further induces vascular damage. Ang-2 controlled by O-GlcNAcylation, transcription factors (TF) and Tie2, might be an important driving force for vascular damage. Glial activation can further contribute to the pathogenesis in hyperglycemia and NDPKB deficiency. Nevertheless, the contribution of the HBP in diabetic neuronal dysfunction still remains unclear. EC: endothelial cell, PC: pericyte, BM: basement membrane, Ang-2: angiopoietin-2, TF: transcription factors.

In hyperglycemia, a relevant amount of the cellular fructose-6-phosphate enters the HBP via the key enzyme GFAT. The activation of the HBP evokes enhanced O-GlcNAc cycling, leading to O-GlcNAc modification of serine or threonine residues of proteins or transcription factors, hence resulting in changes in their function. Hyperglycemia-dependent HBP activation has been linked to the breakdown of the NVU, including damage and loss of pericytes and ECs, glial activation, and neuronal damage in the retina. Interestingly, a similar breakdown of the NVU has been observed in non-hyperglycemic conditions in NDPKB^−/−^ mice (Figure 4). A mechanistic link between the enzymatic function of NDPKB and the activation of the HBP has however not yet been established. Nevertheless, the NDPKB^−/−^ mice exhibit many pathological features also found in DR, such as loss of retinal pericytes and ECs, activation of macro- and microglia, elevation of the protein GlcNAcylation, and alteration in growth factors and transcription factors like Ang-2 and FoxO1. This model provides a possibility for studying common avenues leading to the breakdown of the NVU without secondary effects of diabetes mellitus.

Taken together, the data summarized herein, provide clear evidence that the activation of the HBP and the subsequently altered protein O-GlcNAcylation contribute to the initiation and development of retinopathy. Common features between diabetic and non-diabetic models of the NVU breakdown will likely reveal novel potential targets for the treatment of DR. Nevertheless, more research is required to discover underlying mechanisms.

## Data Availability

Data will be made available on request.
